# Research on dual innovation incentive mechanism in terms of organizations’ differential knowledge absorptive capacity

**DOI:** 10.1371/journal.pone.0256751

**Published:** 2021-08-30

**Authors:** Xuejiao An, Lin Qi, Jian Zhang, Xinran Jiang

**Affiliations:** 1 Beijing Information Science and Technology University, Beijing, China; 2 Beijing World Urban Circular Economy System (Industry) Collaborative Innovation Center, Beijing, China; Shandong University of Science and Technology, CHINA

## Abstract

Differences in the capacity for absorption between different organizations will have an important impact on an organization’s choices of innovation exploration and exploitive innovation strategies. Organizations need to explore correct strategic decisions under different policies for long-term development. This study with limited rational first-mover and late-mover organizations as the research object, based on the evolutionary game theory model, using visualization system deduced first-mover and late-mover organizations in the knowledge absorptive capacity differences and incentive policies under the condition of different strategies selection process. The research shows that the rationality of policy incentive setting has a direct impact on the choice of organizational dual innovation strategy with different knowledge absorption capacities. The market pattern is stable and organizational knowledge absorption capacity is different. The higher the policy incentive level is, the more the organization is inclined to carry out exploratory innovation activities. Under the environment of stable market structure, different organizational knowledge absorption capacity, and no policy incentive, late-mover cannot adopt exploratory innovation strategy alone. When the market pattern is stable and the absorptive capacity of the organization is different, whether the late-mover can adopt the exploratory innovation strategy depends on the policy incentive level. In this case, the optimal situation is to have the opportunity to change to exploratory innovation at the same time as the first-movers.

## Introduction

March proposed the concepts of exploratory learning and exploitative learning in 1991, which were later applied by scholars in the field of innovation. According to the degree of innovation and knowledge base, innovation is divided into two types: exploratory innovation and exploitative innovation [1,2]. Exploratory innovation is radical innovation. It provides new designs, creates new markets, and develops new distribution channels. At the same time, new knowledge or deviation from existing knowledge is required. Exploitative innovation is a gradual innovation that aims to meet the needs of existing customers or the market, improve established designs, expand existing products and services, and improve the efficiency of existing distribution channels. It is an expansion of existing knowledge and skills [3]. Due to the constraints of resource allocation, it is difficult for organizations to carry out dual innovation at the same time [4]. The government can use means such as financial subsidies or tax incentives to encourage enterprises to innovate and effectively alleviate the problem of resource constraints [5,6]. As a driving force for changes in the external environment, policy incentives have an important impact on corporate innovation activities. On the one hand, government subsidies can make up for "market failures", reduce R&D costs, and at the same time release positive signals for corporate development, and drive companies to invest in more organizational innovation resources, resulting in a leverage effect; on the other hand, government subsidies will have leverage on corporate innovation. Input produces a crowding-out effect [7,8]. Companies that receive government subsidies invest more in innovation than those that have not received subsidies [9]. Government subsidies promote the dual innovation of enterprises in different ways [10]. Government subsidies help the rapid application of innovative projects to the market and improve the short-term performance of enterprises by accelerating the speed of utilization innovation; government subsidies promote the long-term development of enterprises by reducing exploratory innovation R&D costs and attracting external financing [11].

Regarding the application of the policy incentive game model, Spencer and Brander conducted groundbreaking research [12], and more scholars subsequently explored this field. Liu Zhiyong’s analysis based on evolutionary game shows that the stable strategy of local government and enterprise cooperative innovation is difficult to realize spontaneously, and the active strategic choice of local government plays an important role in it. At the same time, the further optimization and improvement of the incentive and restraint mechanism is the key to the realization of cooperative innovation of the "dual main body" [13]. Li Wenjian et al. [14] aimed at the stability of new energy vehicle upstream and downstream companies’ cooperative innovation, constructed an evolutionary game model with vehicle companies and battery companies as the main body. Simulation analysis found that the policy-guided market can effectively guide upstream and downstream companies. Cooperative innovation, R&D subsidies, and appropriate tax incentives are more conducive to enterprise cooperative innovation. Shang Bo et al. [15] used evolutionary game theory to focus on the process of selecting two green technological innovation models for resource capture and value creation under different incentive policies. Sun Zhenqing et al. [16] constructed an evolutionary game model for the product selection of duopoly enterprises. Under different levels of environmental taxes and green innovation subsidies, they simulated the dynamic selection process of enterprise production strategies through numerical simulation. Lin Chengliang and Xu Weimin found through constructing a game model that the effect of innovation subsidies is also closely related to the knowledge absorption capacity of enterprises [17].

Knowledge absorptive capacity is the internal motivation of enterprise innovation, and the combination of knowledge and innovation has become the source of power for enterprise development [18]. Absorptive capacity refers to the organization’s ability to recognize, digest, evaluate and commercialize new external knowledge [19,20]. Exploratory innovation is the process of surpassing the existing knowledge base to produce new products and new technologies. Only when the absorptive capacity of the organization reaches a certain level can the transformed knowledge be effectively used [21]. Organizations with strong knowledge absorptive capacity can quickly identify, acquire and use complementary knowledge and resources that they lack within the cooperation network and transform them into the organization’s internal R&D and innovation process based on digestion and absorption, accelerating the innovation process and improving organizational performance [22]. Knowledge absorptive capacity has become the main driving force for competition among R&D-intensive enterprises [23]. Organizations with strong knowledge absorptive capacity are easy to achieve higher innovation performance, while organizations with weak knowledge absorptive capacity are difficult to achieve better innovation performance [19].

In summary, the current research has the following problems: (1) The above-mentioned research provides an important basis for the choice of innovative models of enterprises under different government incentive policies, but these studies ignore the differences in knowledge absorption capacity between enterprises. (2) Considering the degree of rationality of the enterprise itself, and the uncertainty of enterprise innovation activities due to the influence of various factors, it is more reasonable to analyze enterprise innovation behavior under the assumption of bounded rationality. Because of the above analysis, this study introduces the difference in absorptive capacity and government subsidies into the evolutionary game model. The purpose is to analyze the impact of policy factors directly related to the choice of innovation mode in the case of differences in organizational knowledge absorptive capacity. This research adopts the means of dynamic simulation, the evolutionary game model is established based on evolutionary game rules, first-mover organization and late-mover organization are defined as two sides of bounded rationality game, gives organizations within individual autonomous learning and the ability to choose, intuitive display organization innovation strategy evolution process under the government incentives, finding the best government incentive policies, guide enterprises to carry out innovation activities.

## Methods

### Problem description

Dual innovation is an effective means for companies to obtain and maintain competitive advantages, and its formation is a complex causal interaction process [10]. From the perspective of resource demand, exploratory innovation requires companies to obtain new resources, while utilization innovation requires companies to focus on the development of current resources. From the perspective of market demand, utilization innovation generally meets the needs of the public and is positioned at a low level. In the end market, exploratory innovation generally meets the needs of a small number of people and is positioned in the high-end market. [24] Resource conflict is the main contradiction of the imbalance of dual innovation by enterprises [25]. The government’s subsidies for innovation inputs can effectively encourage the organization of innovation activities, change the composition of innovation activities, and ease the imbalance of innovation caused by resource constraints [26]. Literature [13–15] and [16] use evolutionary game theory to carry out relevant research on government incentive policies, which verifies the applicability and feasibility of this method in this type of problem and provides a model basis for the study of this article.

According to the theory of an evolutionary game, the first-mover and late-mover organizations are defined as two parties with bounded rationality. When the differences in absorptive capacity between different organizations are taken into account, organizations with strong knowledge absorptive capacity can take advantage of new knowledge more quickly and conduct exploratory innovation to increase organizational performance. Motivated by government incentive policies, individuals who advocate exploratory innovation within late-mover organizations will support modifications in practices and disturb the stability of the system. Because individuals with bounded rationality cannot accurately calculate their returns, they can only choose to learn from individuals who have obtained high returns before finally reaching a stable equilibrium. With different strategic choices between first-mover and late-mover organizations, the payment income matrix is also different, and the system will eventually reach different stability. This stability creates a point where the optimal choice for the government is the adoption of incentive policies as a consequence of the differences in the knowledge absorptive capacity of organizations.

### Basic hypotheses

In this study, the research objects are the first-mover and late-mover organizations with bounded rationality. Through the method of the evolutionary game, the process of choosing a dual innovation strategy for different organizations under different incentive policies and knowledge absorptive capacities is simulated to find out the best government decision for incentive policies.

Hypothesis 1: This study takes first-mover and late-mover organizations as the research objects. It is assumed that each participating subject is an individual organization with bounded rationality, and the information between the participating subjects is not completely symmetrical with each organization having its innovations. When an organization makes decisions, its strategy S = exploratory innovation or exploitative innovation, and each organization makes its decisions based on its benefits in choosing a superior strategy.
Hypothesis 2: Assume that *x* represents those first-mover organizations that choose exploratory innovation while 1−*x* represents those first-mover organizations that choose exploitative innovation. Assume that y represents those late-mover organizations that choose exploratory innovation and 1−y represents those late-mover organizations that choose exploitative innovation.
Hypothesis 3: The government provides tax policy incentives to organizations that adopt exploratory innovation, and with special financial support that builds a platform to promote the integration of industry, university, and research as well as other welfare policies. To simplify the calculation, it is expressed as the economic benefits plus total G.
Hypothesis 4: For this version of the hypothesis assume that there is no difference for products of the low-end market, and their prices are all *P*_1_. The capacity of the low-end market does not change with the number of producers, and their market capacity is set to be *n*. The market capacity of first-mover organizations in the low-end market is assumed to be *n*_1_. The market capacity of late-mover organizations in the low-end market is *n*−*n*_1_. There is no difference in products of the high-end market, and their prices are all *P*_2_. The capacity of the high-end market does not change with the number of producers, and their market capacity is set to be *N*. Assume that the market capacity of first-mover organizations in the high-end market is *N*_1_, and the market capacity of late-mover organizations in the high-end market is set to be *N*-*N*_1_. Among them, *P*_2_>*P*_1_, *n*>*N*.
Hypothesis 5: Both types of organizations conduct product research and development and gain competitive advantages through innovation. Both types of organizations have R&D investment *C* and can use *C* to carry out innovation in different modes.
Hypothesis 6: The formula for organizational absorptive capacity is *AC*_*i*_ = *δ*_*i*_, assume that the knowledge absorptive capacity of the first-mover organizations is *δ*_1_, the knowledge absorptive capacity of late-mover organizations is *δ*_2_, *δ*_1_>*δ*_2_.
Hypothesis 7: *S* means that one type of organization conducts exploratory innovation activities, and the other type of organization conducts exploitative innovation activities. The organization that adopts exploitative innovation obtains the extra benefits of technology spillover, which is related to the organization’s knowledge absorption capacity.
Hypothesis 8: Suppose that the excess income obtained by the organization using exploratory innovation is Δ*π*.
Hypothesis 9: Let *α* be the profit distribution coefficient of exploratory innovation by the first-mover organizations, and (1−*α*) be the profit distribution coefficient of exploratory innovation by the late-mover organizations.

The terms contained in the above hypotheses are summarized in Table 1 below.

**Table 1:** Symbols and descriptions.

Symbols	Meaning and Descriptions
*x*	The proportion of individuals in first-mover organizations that adopt exploratory innovation, while the proportion of individuals in such organizations that focus on exploitive innovation is 1−*x*
y	The proportion of individuals in late-mover organizations that adopt exploratory innovation, while the proportion of individuals in such organizations that focus on exploitive innovation is 1−*y*
*δ*_1_	Knowledge absorptive capacity of first-mover organizations
*δ*_2_	Knowledge absorptive capacity of late-mover organizations, *δ*_1_>*δ*_2_
*P*_1_	The market price of low-end products
*P*_2_	The market price of high-end products, *P*_2_>*P*_1_
*N*	Market capacity of the high-end market
*N*_1_	Market capacity of first-mover organizations in the high-end market
*n*	Market capacity of the low-end market
*n*_1_	Market capacity of first-mover organizations in the low-end market
*C*	The cost of the organization’s R&D investment, which can be used to innovate in different modes
*S*	One type of organization conducts exploratory innovation activities, and the other type of organization conducts exploitative innovation activities, adopting exploitative innovation organizations to obtain additional benefits from technology spillovers.
Δ*π*	Excessive income obtained by the organization using exploratory innovation
*α*	The benefit distribution coefficient of exploratory innovation for first-mover organizations, and (1−*α*) is the benefit distribution coefficient of exploratory innovation by late-developing organizations.
*G*	Financial subsidies of the government on enterprises that adopt exploratory innovation

## Model construction and analyses

### Model construction

Based on the above hypotheses, establish the evolutionary game payment income matrix of exploratory innovation and exploitive innovation strategy selection for first-mover and late-mover organizations, as shown in Table 2.

**Table 2:** Evolutionary game payment matrix.

Strategy and benefits		Late-mover	
		Exploratory innovation/*y*	Exploitive innovation/1−y
First-mover	Exploratory innovation/*x*	*P*_2_*N*_1_−*C*+*G*+*αΔπ* *P*_2_(*N*−*N*_1_)−*C*+*G*+(1−*α*)Δ*π*	*P*_2_*N*−*C*+*G*+Δ*π* *P*_1_*n*−*C*+*Sδ*_2_
	Exploitive innovation/1−*x*	*P*_1_*n*−*C*+*Sδ*_1_ *P*_2_*N*−*C*+*G*+Δ*π*	*P*_1_*n*_1_−*C* *P*_1_(*n*−*n*_1_)−*C*

If the return of exploitive innovation and exploratory innovation adopted by first-mover organizations is *U*_11_, *U*_12_, and the expected return is *U*_1_, then, the return of the exploitive innovation adopted by first-mover organizations is:

$$U_{11}=y(P_{1}n-C+S\delta_{1})+(1-y)(P_{1}n_{1}-C)$$ (1)

The return of exploratory innovation:

$$U_{\mathrm{12}}=y(P_{2}N_{1}-C+G+\alpha \Delta \pi )+(1-y)(P_{2}N-C+G+\Delta \pi )$$ (2)

The expected return:

$$U_{1}=xU_{12}+(1-x)U_{11}$$ (3)

If the return of exploitive innovation and exploratory innovation adopted by late-mover organizations is *U*_21_, *U*_22_, and the expected return is *U*_2_, then, the return of the exploitive innovation adopted by late-mover organizations is:

$$U_{\mathrm{21}}=x(P_{1}n-C+S\delta_{2})+(1-x)[P_{1}(n-n_{1})-C]$$ (4)

The return of exploratory innovation:

$$U_{22}=(1-x)(P_{2}N-C+G+\Delta \pi )+x[P_{2}(N-N_{1})-C+G+(1-\alpha )\Delta \pi ]$$ (5)

The expected return:

$$U_{2}=yU_{22}+(1-y)U_{21}$$ (6)

Based on the evolutionary game theory, the replication dynamic equation for the dual innovation’s evolutionary game of first-mover and late-mover organizations can be obtained:

$$F_{1}(x,y)=\frac{dx}{dt}=x(1-x)(U_{12}-U_{11})=x(1-x)[\begin{array}{l} (1-y)(P_{2}N+G+\Delta \pi -P_{1}n_{1})+\\ y(P_{2}N_{1}+G+\alpha \Delta \pi -P_{1}n-S\delta_{1}) \end{array}]$$ (7)

$$F_{2}(x,y)=\frac{dy}{dt}=y(1-y)(U_{22}-U_{21})=y(1-y)[\begin{array}{l} (1-x)[P_{2}N+G+\Delta \pi -P_{1}(n-n_{1})]+\\ x[P_{2}(N-N_{1})+G+(1-\alpha )\Delta \pi -P_{1}n-S\delta_{2}] \end{array}]$$ (8)

Let *dx*/*dt* = 0, *dy*/*dt* = 0, and then by solving the replication dynamic equation, we can get five local equilibrium points, namely: (0, 0) (0, 1), (1, 0), (1, 1), (x*,y*) where:

$$x^{*}=\frac{P_{2}N+G+\Delta \pi -P_{1}(n-n_{1})}{P_{1}n_{1}+S\delta_{2}+P_{2}N_{1}+\alpha \Delta \pi }$$ (9)

$$y^{*}=\frac{P_{2}N+\Delta \pi +G-P_{1}n_{1}}{P_{2}(N-N_{1})+(1-\alpha )\Delta \pi +P_{1}(n-n_{1})+S\delta_{1}}$$ (10)

### Equilibrium points and stability analysis

Based on the local stability analysis method proposed by Friedman [27], the local stability for the Jacobian matrix in the system is used to analyze whether the above equilibrium point is the evolutionarily stable strategy of the system.


$$J=(\begin{array}{l} \frac{\partial F_{1}}{\partial x}\frac{\partial F_{1}}{\partial y}\\ \frac{\partial F_{2}}{\partial x}\frac{\partial F_{2}}{\partial y} \end{array})=(\begin{array}{l} f_{11}f_{12}\\ f_{21}f_{22} \end{array})$$ (11)

$$DetJ=|\begin{array}{cc} \frac{\partial F_{1}}{\partial x} & \frac{\partial F_{1}}{\partial y}\\ \frac{\partial F_{2}}{\partial x} & \frac{\partial F_{2}}{\partial y} \end{array}|=|\begin{array}{cc} f_{11} & f_{12}\\ f_{21} & f_{22} \end{array}|=f_{11}\cdot f_{22}-f_{12}\cdot f_{21}$$ (12)

$$f_{11}=(1-2x)[\begin{array}{l} (1-y)(P_{2}N+G+\Delta \pi -P_{1}n_{1})+\\ y(P_{2}N_{1}+G+\alpha \Delta \pi -P_{1}n-S\delta_{1}) \end{array}]$$ (13)

$$f_{12}=-x(1-x)[(1-\alpha )\Delta \pi +P_{1}(n-n_{1})+P_{2}(N-N_{1})+S\delta_{1}]$$ (14)

$$f_{\mathrm{21}}=-y(1-y)[P_{1}n_{1}+P_{2}N_{1}+\alpha \Delta \pi +S\delta_{2}]$$ (15)

$$f_{22}=(1-2y)[\begin{array}{l} (1-x)[P_{2}N+G+\Delta \pi -P_{1}(n-n_{1})]+\\ x[P_{2}(N-N_{1})+G+(1-\alpha )\Delta \pi -P_{1}n-S\delta_{2}] \end{array}]$$ (16)

To facilitate the discussion of the global equilibrium of the local equilibrium points, the transition parameters *TA*1, *TA*2, *TB*1, *TB*2 are introduced to simplify the above formulas, where:

$$TA1=P_{2}N+\Delta \pi +G-P_{1}n_{1}$$ (17)

$$TA2=P_{2}N_{1}+\alpha \Delta \pi +G-P_{1}n-S\delta_{1}$$ (18)

$$TB1=P_{2}N+G+\Delta \pi -P_{1}(n-n_{1})$$ (19)

$$TB2=P_{2}(N-N_{1})+G+(1-\alpha )\Delta \pi -P_{1}n-S\delta_{2}$$ (20)

$$x^{*}=\frac{P_{2}N+G+\Delta \pi -P_{1}(n-n_{1})}{P_{1}n_{1}+S\delta_{2}+P_{2}N_{1}+\alpha \Delta \pi }=\frac{TB1}{TB1-TB2}$$ (21)

$$y^{*}=\frac{P_{2}N+\Delta \pi +G-P_{1}n_{1}}{P_{2}(N-N_{1})+(1-\alpha )\Delta \pi +P_{1}(n-n_{1})+S\delta_{1}}=\frac{TA1}{TA1-TA2}$$ (22)

Since

$$\begin{array}{l} TA1-TA2=P_{1}(n-n_{1})+S\delta_{1}+P_{2}(N-N_{1})+(1-\alpha )\Delta \pi >0\\ TB1-TB2=P_{1}n_{1}+P_{2}N_{1}+\alpha \Delta \pi +S\delta_{2}>0\\ TA1-TB2=S\delta_{2}+P_{2}N_{1}+\alpha \Delta \pi +P_{1}(n-n_{1})>0, \end{array}$$ (23)

is always established. Therefore, *TA*1>*TA*2, *TB*1>*TB*2, *TA*1>*TB*2 is always established. The specific values of local equilibrium points f11, f12, f21, and f22 are listed in Table 3.

**Table 3:** The value of the Jacobian determinant at the local equilibrium point.

*x*,*y*	*f*_11_	*f*_12_	*f*_21_	*f*_22_
(0,0)	*T*_*A*1_	0	0	*T*_*B*1_
(0,1)	*T*_*A*2_	0	0	−*T*_*B*1_
(1,0)	−*T*_*A*1_	0	0	*T*_*B*2_
(1,1)	−*T*_*A*2_	0	0	−*T*_*B*2_
(x*,y*)	0	$\frac{T_{B1}\cdot T_{B2}(T_{A1}-T_{A2})}{{\left(T_{B1}-T_{B2}\right)}^{2}}$	$\frac{T_{A1}\cdot T_{A2}(T_{B1}-T_{B2})}{{\left(T_{A1}-T_{A2}\right)}^{2}}$	0

If and only if 0<x*<1 and 0<y*<1, there are five local equilibrium points in the system, namely:

$$0<\frac{TB1}{TB1-TB2}<1$$ (24)

$$0<\frac{TA1}{TA1-TA2}<1$$ (25)

By simplification, it is obtained that: *TA*1×*TA*2<0, *TB*1×*TB*2<0. That is, when *TA*1 and *TA*2, *TB*1 and *TB*2 have different signs, there are five equilibrium points in the system. Otherwise, there is no local equilibrium point. *TA*1>*TA*2, *TB*1>*TB*2, *TA*1>*TB*2 is always established. In the model construction of this study, therefore, when the evolutionarily stable strategy is discussed, there is no *TA*1<0 and *TA*2>0, neither is there *TB*1<0 and *TB*2>0, and neither is there *TA*1<0 and *TB*2>0. When the determinant of the Jacobian row matrix corresponding to the local equilibrium point is DetJ>0, and its trace is TrJ<0, the local equilibrium points gradually converge and tend to be stable, and the corresponding strategy becomes the evolutionary stable strategy (ESS). When the parameter value range that does not meet the actual situation is excluded, the transition parameter values that meet the conditions are shown in Table 4.

**Table 4:** Analysis of evolutionary stability strategy.

	Transition parameter				(0,0)			(0,1)			(1,0)			(1,1)			(x*,y*)		
	TA1	TA2	TB1	TB2	DetJ	TrJ	Type	DetJ	TrJ	Type	DetJ	TrJ	Type	DetJ	TrJ	Type	DetJ	TrJ	Type
1	+	-	+	-	+	+		+	-	ESS	+	-	ESS	+	+		-	0	
2	+	+	+	-	+	+		-			+	-	ESS	-					
3	+	+	+	+	+	+		-			-			+	-	ESS			
4	+	-	+	+	+	+		+	-	ESS	-			-					
5	+	+	-	-	-			+	+		+	-	ESS	-					
6	-	-	+	-	-			+	-	ESS	-			+	+				
7	+	-	-	-	-			-			+	-	ESS	+	+				
8	-	-	-	-	+	-	ESS	-			-			+	+				

Note: Only the transition parameter value corresponding to sequence number 1 has a local equilibrium point (x*,y*).

Based on Table 4, it is known that:

Conclusion 1: Whether the local equilibrium point is the global equilibrium point is not affected by the proportion x, y when individuals choose either exploratory innovation strategies or exploitive innovation strategies in the first-mover and late-mover organizations.
Conclusion 2: If a local equilibrium point (x*,y*) exists, then the trace at this point is always 0; that is, TrJ = 0 is always established. Therefore, (x*,y*) is not a global equilibrium point.
Conclusion 3: When *TA*1<0 and *TB*1<0, namely, *G*<min[*P*_1_*n*_1_−*P*_2_*N*−Δ*π*,*P*_1_(*n*−*n*_1_)−*P*_2_*N*−Δ*π*], when the two types of organizations adopt exploratory innovation after deducting R&D costs, there is no advantage compared with the use of utilization innovation. Neither the first-mover organization nor the late-mover organization conducts exploratory innovation activities. (0, 0) is the only evolutionary stable point of the system.
Conclusion 4: When *TA*1>0 and *TB*2<0, namely, *P*_1_*n*_1_−*P*_2_*N*−Δ*π*<*G*<*P*_1_*n*+*Sδ*_2_−*P*_2_(*N*−*N*_1_)−(1−*α*)Δ*π*, for the first-mover organization, when the late-mover organization chooses the exploitive innovation strategy, since *TA*1>0, it chooses exploratory innovation to get more profits; for the late-mover, the first-mover organization chooses exploratory innovation, because *TB*2<0, it means that one’s own choice of exploitive innovation is more advantageous, and the two parties constitute an equilibrium state on the pure strategic combination (1, 0).
Conclusion 5: When *TA*2<0 and *TB*1>0, namely, *P*_1_(*n*−*n*_1_)−*P*_2_*N*−Δ*π*<*G*<*P*_1_*n*+*Sδ*_1_−*P*_2_*N*_1_−*α*Δ*π*, when late-mover organizations choose exploratory innovation strategies, because *T*_*A*2_<0, it is more advantageous for first-mover organizations to choose exploitive innovation strategies. Therefore (0, 1) is the only evolutionary stable point of the system.
Conclusion 6: When *TA*2>0 and *TB*2>0, namely, *G*>max[*P*_1_*n*+*Sδ*_1_−*P*_2_N_1_−*α*Δ*π*,*P*_1_*n*+*Sδ*_2_−*P*_2_(N−*N*_1_)−(1−*α*)Δ*π*], for the first-mover organization, when the late-mover organization chooses exploratory innovation, because *T*_*A*2_>0, the first-mover organization chooses the exploratory innovation strategy to make more profits; for the late-mover organization, when the first-mover organization chooses the exploratory innovation strategy, because *TB*2>0, the late-mover organization proceeding from rationality, an exploratory innovation strategy should be selected, so (1,1) constitutes the only Nash equilibrium of the system.
Conclusion 7: When *TA*1>0, *TA*2<0, *TB*1>0 and *TB*2<0, namely, $\begin{array}{l} \max[P_{1}n+S\delta_{1}-P_{2}N_{1}-\alpha \Delta \pi ,P_{1}n+S\delta_{2}-P_{2}(N-N_{1})-(1-\alpha )\Delta \pi ]\\ <G<\min[P_{1}n_{1}-P_{2}N-\Delta \pi ,P_{1}(n-n_{1})-P_{2}N-\Delta \pi ] \end{array}$, for the first-mover organization, when the late-mover organization chooses the exploitive innovation strategy, since *TA*1>0, the first-mover organization chooses the exploratory innovation strategy to gain more profits; when the late-mover organization chooses the exploratory innovation strategy, because *TA*2<0, the first-mover organizations have more advantages in choosing exploitive innovation strategies. Therefore, the system has two evolutionary stable strategies (0,1) and (1,0).

## Results and discussion

To verify the reliability of the previous theoretical analysis, NetLogo software is used to construct a simulation model and perform an evolutionary game simulation analysis. The model assumes that the number of subjects is 200, where 20% are first-mover organizations and 80% are late-mover organizations. Heterogeneous subjects play repeated games in the random walk network space. Heterogeneous subjects accumulate returns through games, and homogenous subjects adopt an updated strategy of simulating the optimal rules. To simulate the bounded rationality of the strategy updates caused by the limited information, limited logical reasoning ability, emotional fluctuations, and other factors in the real world, the Fermi dynamics process is adopted in the strategy update. That is, among the strategies,

$$\varphi \left(s_{i}\leftarrow s_{j}\right)=\frac{1}{1+\exp[\eta (P_{i}-P_{j})]}$$ (26)

*s*_*i*_、 *s*_*j*_ are the strategies of the subjects *i* and *j*, respectively. *φ*(*s*_*i*_←*s*_*j*_) is the probability that the subject *i* adopts the subject *j*’*s* strategy. *P*_*i*_、*P*_*j*_ are the returns of subject *i* and subject *j*, respectively. *η* is the degree of irrationality, where the larger the value *η*, the greater the probability of the high-return strategy being imitated, and the smaller the value *η*. The simulation of high-return strategies is completely random.

Refer to the parameter setting methods and parameter value ranges in [28] and [29] in the literature. The relevant parameter settings are shown in Table 5. The evolutionary game is played until the proportion of the strategy of the system of subjects stabilizes, and individual mutant individuals cannot invade the group. To eliminate randomness, the simulation experiment with each set of parameter settings is repeated 50 times. The result when the strategy evolution trend is the same should prevail.

**Table 5:** Values of evolution simulation parameters.

*x*	y	*δ*_1_	*δ*_2_	*P*_1_	*P*_2_	*n*	*n*_1_	*N*	*N*_1_	*S*	*α*	Δ*π*
0.5	0.5	0.6	0.3	2	4	95	45	15	5	10	0.6	20

### Stability analysis of the system

**Fig 1:**
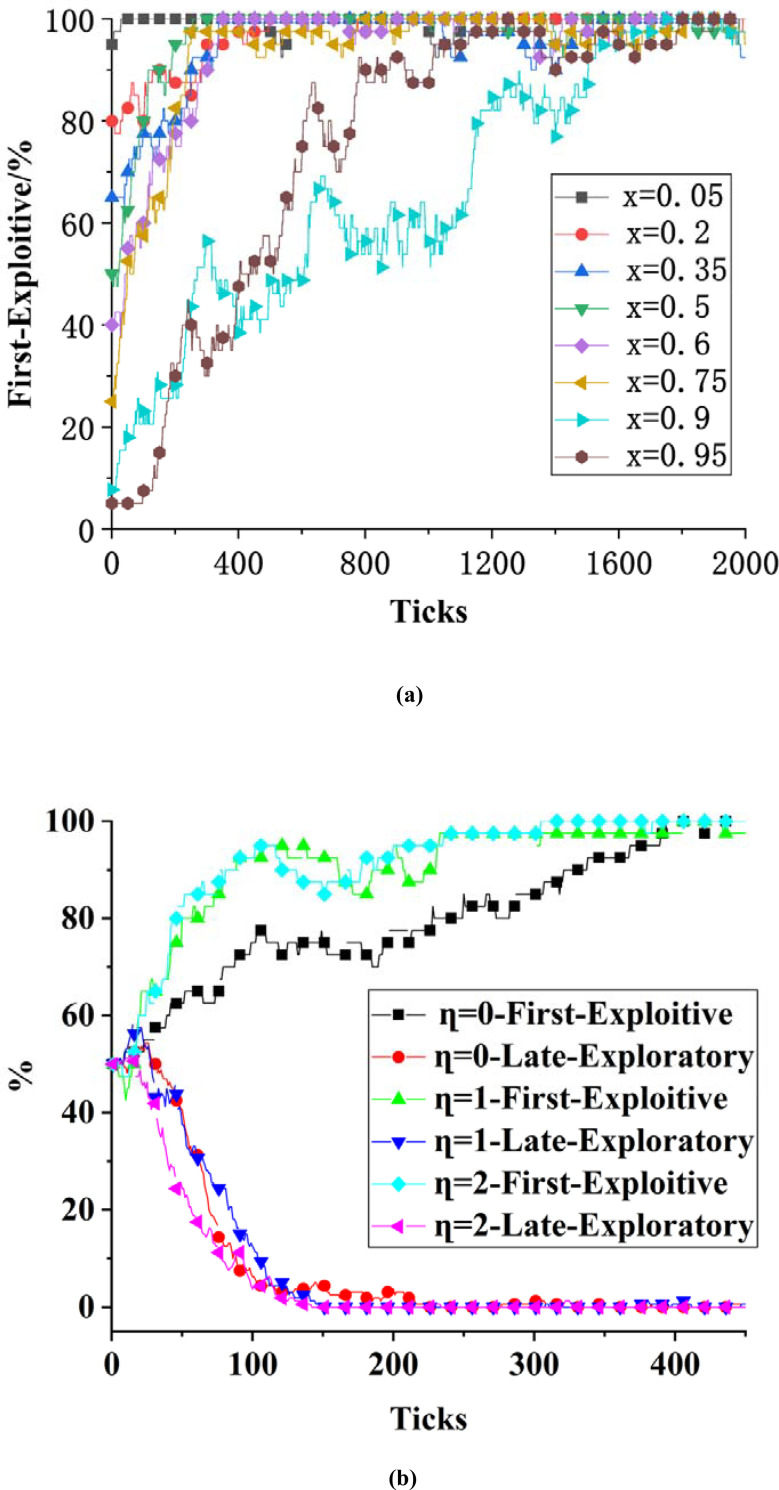
System stability analysis.

To verify the system stability is independent of the values of x and y parameters, the values of the parameter settings are shown in Table 5. It is verified that the values of x, y do not affect the conclusions. The output result is shown in Fig 1(A) when x is used as the independent variable and fixing the values of other parameters remain fixed. Based on the analyses, the proportion of the exploitive innovation strategy adopted initially by different first-mover organizations will affect the system’s tendency to stabilize based on different evolutionary paths. This proportion cannot affect the final stability of the system, indicating that the results for the evolutionary simulation remain consistent with the analysis results (Conclusion 1) of the evolutionarily stable strategy. To verify the impact of irrationality on the system’s stability, set the value of *η* to 0、1、2, and then observe the output results, which are shown in Fig 1(B). The process of game playing in the system is different under different degrees of irrationality, but it does not affect the final stability of the system.

### Simulation of the evolutionary game model with stability

According to the distribution characteristics of ESS in different intermediate variable systems, policy incentive G is taken as an independent variable, the evolutionary game simulation method is used for the analysis, and changes in the system’s stability under five different situations are determined. The parameter settings of the experiment are shown in Table 5, and are used to verify conclusions 3~7.

**Fig 2:**
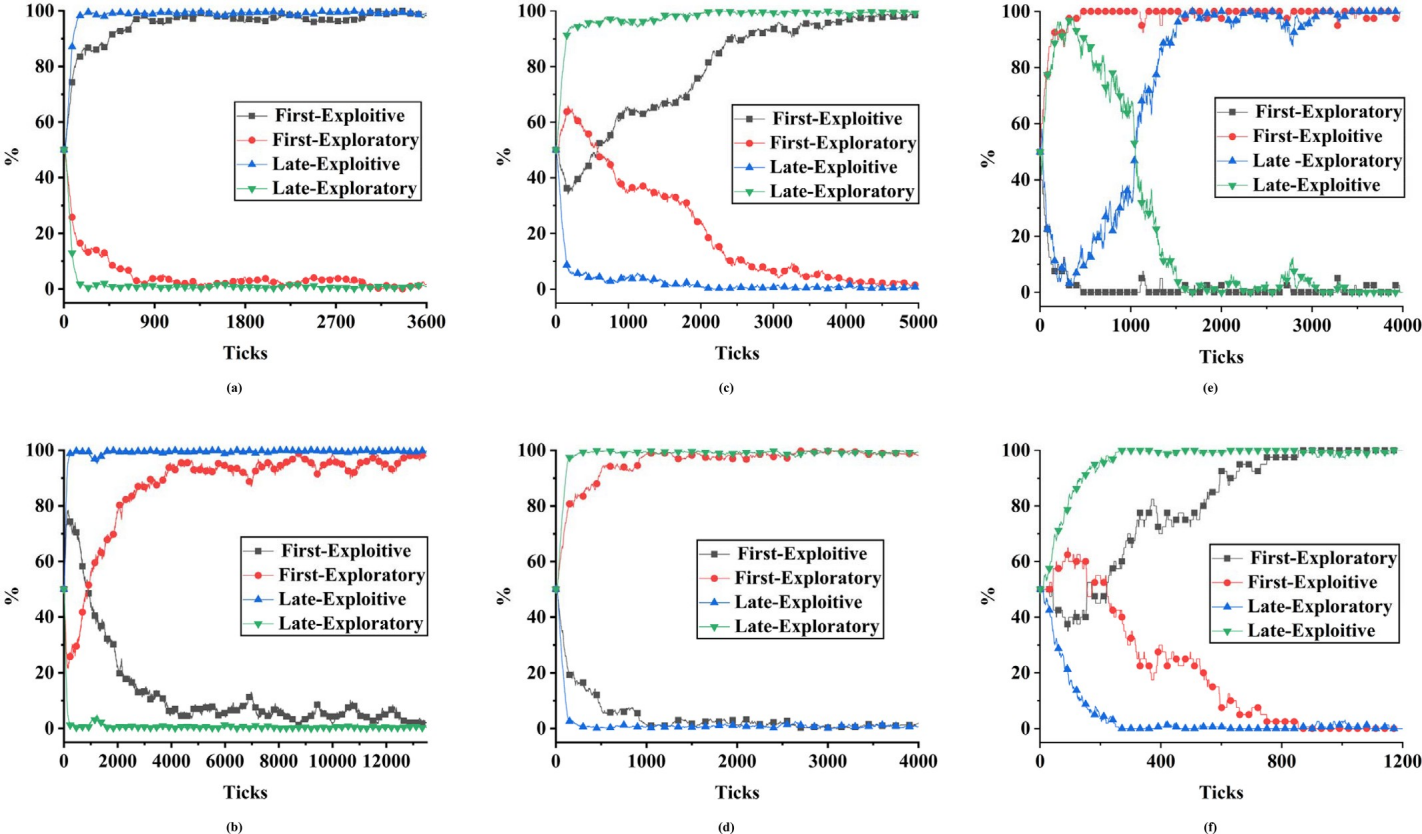
Simulation experiment results of single ESS for the system.

Verification conclusion 3, keep other parameter settings as shown in Table 5, set G = 1. When the market structure is stable and there are differences in organizational absorptive capacity, and the government incentives G is not sufficient to induce any organization to carry out exploratory innovation. If the organization adopts exploratory innovation, it will incur economic losses by itself. The returns obtained are far less than the cost of using exploratory innovation inputs, resulting in a situation where the losses outweigh the gains. In the entire evolutionary game process, a mutant individual advocating exploratory innovation will be generated within the organization, but this mutant individual cannot influence other individuals in first-mover or late-mover organizations, and thus cannot influence the group’s strategy. Due to the bounded rationality of individuals in the organization, individuals who advocate exploratory innovation generated by disturbances will soon be assimilated to the view of other individuals, thereby stabilizing the system at the equilibrium point (0, 0), as shown in Fig 2(A). Conclusion 3 is thus proved.

Verification conclusion 4, keep other parameter settings as shown in Table 5, set G = 18. When the market structure is stable and there are differences in organizational absorptive capacity, the government incentives G is not sufficient to induce exploratory innovation by late-mover organizations. Government incentives can induce exploratory innovation by first-mover organizations to obtain higher returns. Individuals in a first-mover organization that adopt exploratory innovation will use random walks to play games with their opponents. The evolutionary game of the same kind in the group will expand rapidly, and eventually evolve into individuals in first-mover organizations adopting exploratory innovation strategies. As for late-mover organizations, the implementation of exploratory innovation strategies will lead to far fewer returns than the cost involved in adopting exploratory innovation, resulting in a situation where economic losses are not worth the value of the gains. Due to the bounded rationality of individuals in the group, individuals who advocate exploitive innovation approaches in response to disturbances quickly conform to the attitudes of other individuals in the group and become individuals who advocate exploratory innovation. The result is a situation where all first-mover organizations eventually evolve into ones that maintain exploratory innovation strategies, while all late-mover organizations evolve into ones that maintain exploitive innovation strategies, thus reaching stability in the system (1, 0), as shown in Fig 2(B). Conclusion 4 is thereby proved.

Verification conclusion 5, keep other parameter settings as shown in Table 5, set G = 150. When the market structure is stable and there are differences in organizational absorptive capacity, the government incentives G can make the late-mover organizations with weak knowledge absorption ability and poor resource utilization and integration ability carry out exploratory innovation to obtain higher benefits. First-mover organizations have a relatively strong knowledge absorptive capacity, and if they adopt exploratory innovation strategies, they will incur economic losses. Since late-mover organizations have relatively weak knowledge absorptive capacity, those individuals in the group that utilizes exploratory innovation will expand rapidly through random walks to play games with their opponents. Eventually, late-mover organizations will all adopt exploratory innovation strategies to achieve stability in the system (0, 1), as shown in Fig 2(C). Conclusion 5 is thus proved.

Verification conclusion 6, keep other parameter settings as shown in Table 5, set G = 200. When the market structure is stable and there are differences in organizational absorptive capacity, the government incentives G will induce both first-mover and late-mover organizations to carry out exploratory innovation. Regardless of whether the ratio of input to output within the organization is greater than 1, policy rewards can make the returns of an organization adopting exploratory innovation far greater than the cost of adopting exploratory innovation for an organization. Encouraged by internal incentive policies, the organization eliminates all individuals who transform from mutation to adopting exploitive innovation so that all individuals within the organization implement exploratory innovation strategies, and tend to stabilize until stability is reached as shown in Fig 2(D). Conclusion 6 is thus proved.

Verification conclusion 7, keep other parameter settings as shown in Table 5, set G = 50, P_1_ = 2, P_2_ = 2.4. When the price difference between the high-end market and the low-end market is very small and there is a difference in the absorptive capacity of the organization, the government incentives G is not sufficient to induce both first-mover and late-mover organizations to conduct exploratory innovation at the same time. Only one organization can profit from adopting exploratory innovation. With multiple experimental simulations under the same parameters, the probability of “first-mover organizations adopting exploitive innovation, late-mover organizations adopting exploratory innovation” (Fig 2(E)) remains the same as the probability of “first-mover organizations adopting exploratory innovation, late-mover organizations adopting exploitive innovation” (Fig 2(F)). The result is not affected by the initial proportion of individuals and strategies. Conclusion 7 is thereby proved.

## Conclusions

Based on the assumption of differences in organizational absorptive capacity, this paper constructs a dualistic innovation strategy choice game model with bounded rationality based on evolutionary game theory, starts with government differentiated incentive policies, considers the choice of organizational innovation strategies under different policy conditions, and adopts Visual simulation simulates the influence of differentiated policy incentives on the evolution path of system simulation. The research demonstrates that, in addition to organizations being restricted by their knowledge absorptive capacity, government incentive policies play a decisive role in the choice of innovation strategies. The specific research conclusions are as follows:

1. Because the limited innovation resources will prompt organizations to choose between two innovations [30], the higher the level of policy incentives, the more inclined organizations are to carry out exploratory innovation activities.
2. Only when the policy incentives meet the profitability conditions of the organization adopting exploratory innovation will the final stability of the system transform from exploitive innovation to exploratory innovation. When policy incentives do not meet the profitability conditions of the organization adopting exploratory innovation, the path to stability will become more volatile, but the final stability result can never be changed. After the organization makes a profit from conducting exploratory innovation, the evolutionary path will converge much faster until stability is reached if the degree of policy incentives is still increasing.
3. When policy incentives G is not sufficient to induce both first-mover and late-mover organizations to carry out exploratory innovation at the same time, the only condition satisfied is only one type of organization adopts exploratory innovation to make profits, and the system’s evolution will give rise to two types of stability where the strategy is either “first-mover organizations adopting exploitive innovation” or “first-mover organizations adopting exploratory innovation”.
4. When the market structure is stable, the absorptive capacity of the organization is different, and there is no policy incentive environment, it is impossible for late-mover organizations to independently adopt exploratory innovation strategies. When the market structure is stable, the absorptive capacity of the organization is different, whether late-mover organizations can adopt exploratory innovation strategy depends on the level of policy incentives, and in this case, the best opportunity for late-mover organizations is to simultaneously turn to exploratory innovation with first-mover organizations.
5. In an environment with a stable market structure, if the late-mover organization adopts exploratory innovation alone, it will catch up with the knowledge boundary of the first-mover organization, except for factors such as the very small price difference between the high-end market and the low-end market and the low level of policy incentives. In addition to the unprofitable use of exploratory innovation by the first-mover organizations, it is more important that the late-mover organizations themselves take the initiative to reduce the difference in knowledge absorption capacity between the first-mover organizations and the first-mover organizations.

Based on the above conclusions, the following recommendations are proposed:

1. Policymakers must always pay attention to the impact of incentive policies on organizations with different knowledge absorptive capacity, and formulate incentive policies that are in line with organizational development. In addition, incentive policies must be targeted, formulate special policies that promote the improvement of absorptive capacity, and highly stimulate organizational knowledge absorption Improved ability to improve innovation performance [31].
2. Organizations with differences in knowledge absorptive capacity should take into account their knowledge absorptive capacity, R&D level, and profits, and consider policy incentives in a targeted manner. For first-mover organizations, which have the advantage of strong knowledge absorption capacity and can use the technology spillover activities of exploratory innovation organizations to obtain benefits. Therefore, they must selectively carry out exploratory innovation activities to maximize their interests. For late-mover organizations, it is necessary to seize the opportunity of the government’s incentive policies, carry out exploratory innovation, and gradually narrow the gap with the first-mover organizations to make profits.

Our research elaborates on the evolutionary influence of policy incentives on the choice of dual innovation strategies in organizations when there are differences in the knowledge absorptive capacity of organizations. The model constructed above is universal for the dual innovation strategic decision-making of first-mover and late-mover organizations under conditions of bounded rationality. This study, therefore, is capable of providing a reference point for the government to formulate appropriate incentive policies. This study also has certain limitations as follows:

In this paper, the research on the enterprise innovation incentive mechanism model based on the difference of knowledge absorptive capacity, in the process of practical application, first need to consider whether the market structure is stable; second, whether the innovation activities of enterprises can be successful; third, the absorption of knowledge between enterprises Changes in capacity differences. These are the limitations of the model in practical applications and are also the directions for further research.

## Appendix

### Sensitivity analysis

**Fig 3:**
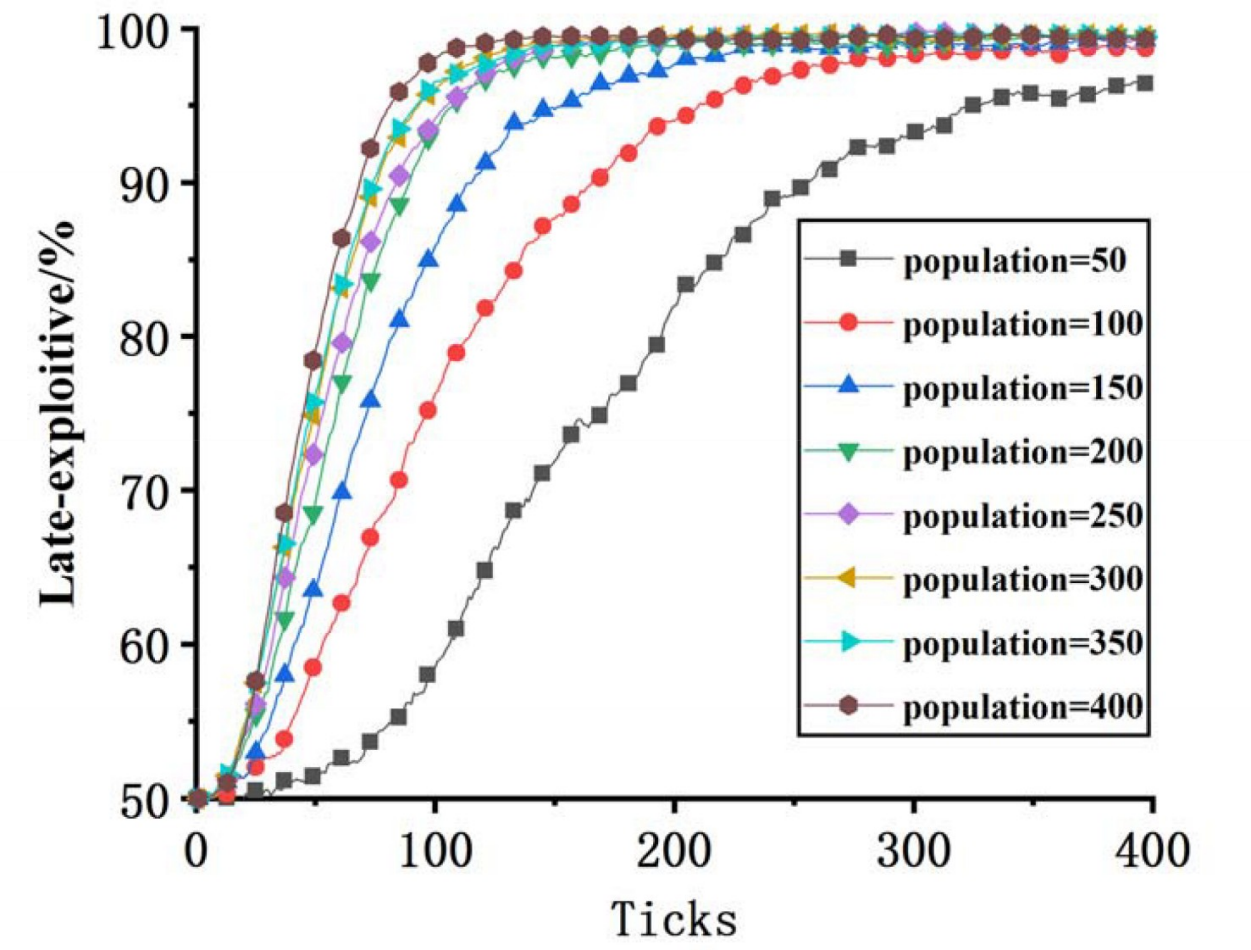
Sensitivity analysis.

Taking the change in the number of subjects in the model set as the independent variable, and the values of other parameters as shown in Table 5, the curve change represents the evolution process of the late-mover organization’s exploitive innovation strategy. It can be seen from Fig 3 that with the increase in the number of entities, the late-mover organization learns from individuals with high returns and choose exploitive innovation strategy faster, but they will eventually reach a stable equilibrium state.
